# The Interplay of Host Autophagy and Eukaryotic Pathogens

**DOI:** 10.3389/fcell.2018.00118

**Published:** 2018-09-13

**Authors:** Robert J. Evans, Varadharajan Sundaramurthy, Eva-Maria Frickel

**Affiliations:** ^1^Host-Toxoplasma Interaction Laboratory, The Francis Crick Institute, London, United Kingdom; ^2^National Centre for Biological Sciences, Tata Institute of Fundamental Research, Bengaluru, India

**Keywords:** autophagy, *Plasmodium*, *Toxoplasma*, *Leishmania*, fungi, pathogenesis, host

## Abstract

For intracellular pathogens, host cells provide a replicative niche, but are also armed with innate defense mechanisms to combat the intruder. Co-evolution of host and pathogens has produced a complex interplay of host-pathogen interactions during infection, with autophagy emerging as a key player in the recent years. Host autophagy as a degradative process is a significant hindrance to intracellular growth of the pathogens, but also can be subverted by the pathogens to provide support such as nutrients. While the role of host cell autophagy in the pathogenesis mechanisms of several bacterial and viral pathogens have been extensively studied, less is known for eukaryotic pathogens. In this review, we focus on the interplay of host autophagy with the eukaryotic pathogens *Plasmodium spp, Toxoplasma, Leishmania spp* and the fungal pathogens *Candida albicans, Aspergillus fumigatus* and *Cryptococcus neoformans*. The differences between these eukaryotic pathogens in terms of the host cell types they infect, infective strategies and the host responses required to defend against them provide an interesting insight into how they respond to and interact with host cell autophagy. Due to the ability to infect multiple host species and cell types during the course of their usually complex lifestyles, autophagy plays divergent roles even for the same pathogen. The scenario is further compounded since many of the eukaryotic pathogens have their own sets of either complete or partial autophagy machinery. Eukaryotic pathogen-autophagy interplay is thus a complex relationship with many novel insights for the basic understanding of autophagy, and potential for clinical relevance.

## Introduction

Most eukaryotic pathogens are characterized by a wonderfully complicated lifestyle often involving serial infection of multiple host organisms from different orders of life and distinct host cell types within a single host organism. Sequential passage through these very different and diverse host cells is thus a central element of their lifestyle. Consequently, they encounter divergent physiological and cellular environments within a particular host, as well as dramatic shifts in these environments as they alter between these host cell types. In addition, several stages represent major amplification steps where the parasite grows in numbers by several logarithmic fold.

**FIGURE 1:**
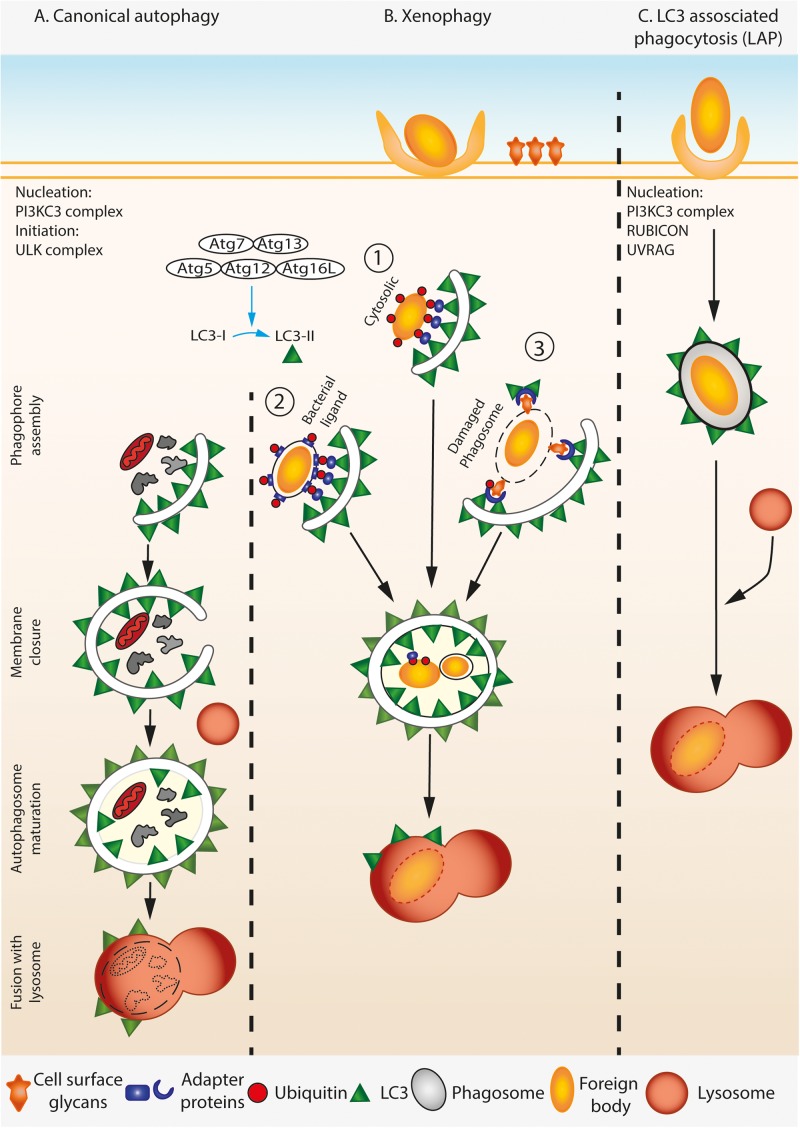
A simplified overview of canonical autophagy, xenophagy, and LAP. **(A)** During canonical autophagy, the nucleation and initiation complexes orchestrate the assembly of phagophore. LC3 is processed and lipidated by covalent conjugation of phosphatidylethanolamine (PE) to its C-terminal Glycine by ATG complex. Lipidated LC3 associates with the growing phagophore membrane by inserting the PE. The growing double membrane engulfs the cytosolic contents including damaged organelle (shown here by mitochondria), protein aggregates, etc., trapping them inside when the membranes seal from the growing ends. The resulting mature autophagosome is double membraned and is marked by LC3-II on both its inner and outer membranes. Mature autophagosome fuse with lysosomes, and the cargo is degraded by the acidic environment of the lysosome. **(B)** During xenophagy, foreign particles in the cell such as invading pathogens are specifically identified by the autophagy machinery. Both cytosolic **(1)** and vacuolar pathogens **(2)** that display a pathogen specific ligand are ubiquitylated and bind to distinct adaptor proteins that can recruit LC3, thereby targeting the pathogen to the autophagosome. Alternatively, host cell can infer the presence of pathogen by detecting the usually cell-surface localized glycans on damaged phagosomal membranes in a ubiquitin dependent manner **(3)**, thus marking them as target for the downstream autophagy machinery. **(C)** Lysosomal associated phagocytosis (LAP) involves association of lipidated LC3 on the cytosolic leaflet of the single membrane phagosome that contains the pathogen. Consequently, LAP does not require some components of the autophagy initiation complex, but needs components of the nucleation complex.

Autophagy is a conserved cell-autonomous catabolic stress response pathway dedicated to the breakdown of cellular material and cell content recycling. Canonical autophagy involves formation of double membrane autophagosomes around the cellular materials to be broken down. The ubiquitin-like machinery, including Atg7 (E1-like), Atg3 (E2-like) and the Atg12-Atg5-Atg16L1(E3-like) complex brings Atg8 proteins such as LC3 to the autophagosome isolation membrane (Mizushima et al., 2002). Membrane-bound LC3 associates with the cargo via autophagy adaptor proteins on the cargo (Randow, 2011; Gomes and Dikic, 2014). Autophagosome membranes surround the cargo and finally deliver it to lysosomes for destruction (**Figure 1A**).

Apart from its homeostatic role, autophagy is actively involved in the clearance of pathogens. However, the role of autophagy during infection is complex, some pathogens rely on induction of host autophagy to survive within host cells while others are destroyed by it (Gomes and Dikic, 2014). As a result, many pathogens have evolved distinct mechanisms to exploit or subvert these pathways. Consequently, the induction of autophagy during intracellular infection can lead to the capture, breakdown and eventual killing of intracellular pathogens, thereby aiding in their detection by the host cell and subsequent activation of the immune response, e.g., via antigen presentation by professional antigen presenting cells. On the other hand, in the same way that autophagy provides nutrients to host cells during starvation, it has the potential to provide nutrients for intruding pathogens.

A specialized form of autophagy called xenophagy involves recognition of the foreign particle or pathogen by host cell receptors, which initiate autophagosome formation and engulfment of the intruding object by a double membrane autophagosome. Xenophagy, generally, is induced by a pathogen or particle found free within the host cell cytosol, or vacuolar pathogen which expresses a pathogen receptor on vacuole membrane, or pathogen residing inside damaged or perforated vacuole (**Figure 1B**). In all these cases, a double membrane autophagosome engulfs the free pathogen or the vacuole containing the pathogen. Another form of autophagy called LC3-associated phagocytosis (LAP) can also be activated during intracellular infection. This non-canonical form of autophagy involves the recruitment of LC3 and other components of the canonical autophagy pathway to foreign particles that are already contained within a single membraned phagosome or endosome (**Figure 1C**). LAP requires some, but not all of the canonical autophagy machinery. The core PI3KC3 complex involved in nucleation (Beclin 1, Atg14L, VPS34, and VPS15), Atg3, Atg4, Atg7, Atg12, Atg16L are required for LAP while the components involved in initiation including the ULK complex (ULK1/2, Atg13, Atg101, and FIP200), ATG14L, WIPI2, and AMBRA1 are not (Funderburk et al., 2010; Martinez et al., 2015; Heckmann et al., 2017; Schille et al., 2017). LAP also requires the proteins Rubicon and UVRAG which are not required for canonical autophagy (Martinez et al., 2015). The end result of this pathway is the deposition of LC3 on the cytosolic side of the single membrane phagosome membrane, which is thought to lead to faster fusion with lysosomes.

The sub-cellular location of the pathogen and the integrity of the vacuole membrane seems to determine mostly whether a pathogen encounters LAP or xenophagy during host cell infection. While xenophagy occurs against pathogens that have invaded the cytosol of host cells either via invasion from the extracellular space and/or following escape from a phagosome, or reside in phagosomes that are damaged or express pathogen derived receptors on vacuolar membrane, LAP is induced against particles that have actively been taken up by host cells via phagocytosis. A further level of complexity is added by apicomplexan parasites such as *Toxoplasma* and *Malaria* spp. that invade host cells using their own machinery, but reside within membrane-enclosed compartments within host cells that have been co-opted by the parasite from the outer membrane during cellular invasion. These atypical compartments known as parasitophorous vacuoles have membranes derived from the host, but contain parasite derived proteins. Due to this they are not treated by host cells in the same way as a phagosome or autophagosome.

In this review, we will highlight the fascinating aspects of autophagy during intracellular *Toxoplasma* and *Plasmodium* spp growth, development and elimination. We will additionally cover current knowledge of the interplay of host autophagy and several species of the parasite *Leishmania*. We do not review limited literature that suggests host autophagy facilitates *Trypanosoma cruzi* invasion and infection (Romano et al., 2009; Vanrell et al., 2013), and whether autolysosomes form around the parasite is contested (Onizuka et al., 2017). To contrast these eukaryotic parasites, we will discuss another group of eukaryotic pathogens from the fungal kingdom and the induction of LAP against them following phagocytosis by host cells. To our knowledge these organisms are the only eukaryotic pathogens with a substantial amount of literature on their interplay with host autophagy.

## Autophagy and *Plasmodium*

Parasites of the genus *Plasmodium* cause malaria, a disease that has left indelible imprints on humanity culturally and genetically, while continuing to have devastating impact in terms of mortality and morbidity, leaving lasting social and economic footprints (Carter and Mendis, 2002; Ashley et al., 2018). During their life cycle, the malarial parasite alternates between the mosquito and mammalian hosts.

### Host Autophagy During the *Plasmodium* Life Cycle – Mosquito Stages

During the mosquito stages, the malarial parasite undergo dramatic and unique changes. Fertilization of the male and female gametocytes in the gut produces the zygote, which is the only diploid phase of the parasite, followed by ookinete stage, which is the only meiotic stage. The highly motile ookinetes cross the gut lining and develop into oocysts while embedded in the extracellular matrix, resulting in the only extracellular developmental stage. Thousands of sporozoites emerge from the oocysts stage and accumulate in the salivary gland, ready to infect a new mammalian host during the next blood meal (Aly et al., 2009). The role of host response and host cellular processes during these transitions is not well explored, although given the largely extracellular nature of these stages, cell-autonomous mechanisms like autophagy might not have a central role.

### Host Autophagy During the *Plasmodium* Life Cycle – Mammalian Stages

When an infected mosquito bites a mammalian host for its blood meal, it injects the infective sporozoites into the skin, from where they home into the liver and infect hepatocytes. Within these cells, the parasites undergo an amplification stage of over 10,000-fold to form merozoites, which egress from the hepatocytes and infect red blood cells. Thus, within mammalian hosts, the parasite encounters two distinct cell types, hepatocytes in the liver and red blood cells in the blood.

#### Host Autophagy During the *Plasmodium* Life Cycle – Red Blood Stages

The red blood cell is devoid of organelles and autophagy processes do not exist. While *Plasmodium falciparum* and *Plasmodium berghei* invade mature red blood cells, other *Plasmodium spp* (notably *Plasmodium vivax* and *Plasmodium yoelii*) invade immature reticulocytes, sequester in the bone marrow (Thomson-Luque and Scopel, 2015) and remodel the reticulocytes. Reticulocyte remodeling, independent of *Plasmodium* infection, is a critical homeostatic process during hematopoiesis, where autophagy plays a key role (Ney, 2011; Griffiths et al., 2012; Mankelow et al., 2016). Defective autophagy during this step results in strong phenotypes such as severe anemia (Mortensen et al., 2010; Mankelow et al., 2016). Interestingly, it has been proposed that *P. vivax* infection triggers, remodels and indeed accelerates the maturation of immature CD71 positive reticulocytes (Malleret et al., 2015) into CD71 negative red blood cells. Hence it is tempting to speculate that *P. vivax* infection could significantly modulate the host cell autophagy during the infection of immature reticulocytes. However, little information is currently available, largely due to the notorious experimental refractoriness of *P. vivax*.

#### Host Autophagy During the *Plasmodium* Life Cycle – Liver Stages

Unlike the mosquito and blood stages, the autophagy machinery of hepatocytes plays a central role in the development of the parasite during the liver stages (Agop-Nersesian et al., 2018). Consequently, the interplay of the host autophagy machinery with the malarial parasite during the liver stage development is an active area of investigation. In this review, we will focus on the role of the hepatocyte autophagy machinery during *Plasmodium* liver stage development.

The *Plasmodium* parasite, during their development within the hepatocytes, is shielded from the host cytosolic defense mechanisms by the parasite vacuole membrane (PVM). The PVM is originally derived from the host cell plasma membrane, but is extensively modified by the parasite, which inserts its proteins to this membrane (Meis et al., 1983; Lingelbach and Joiner, 1998; Nyboer et al., 2017). Some of these proteins are therefore likely to directly interact with cytosolic defense mechanisms and subvert them. Although a handful of such proteins have been characterized, molecular functions have been ascribed to only a few of them. Most interestingly, mutants for some of the proteins such as UIS3, UIS4 result in growth arrest of the parasite (Mueller et al., 2005), suggesting their essential function during the liver stage.

Recent years have seen rapid advancement in the knowledge on the interaction of liver stage *Plasmodium* with the host cell autophagy machinery. While the liver stage has been traditionally termed as the “silent stage” of malaria, it is now becoming clear that the host cell indeed senses the parasite and responds accordingly. In fact, many parasites are eliminated by the host cell defense mechanism during *Plasmodium* liver stage development, with autophagy playing a key role (Schmuckli-Maurer et al., 2017). Induction of canonical non-selective autophagy supports parasite development in hepatocytes, as starvation or Rapamycin treatment resulted in an increase in the number of liver stage parasites (Prado et al., 2015; Zhao et al., 2016). Similarly, parasite development is affected by genetic abrogation of the host autophagy machinery (Prado et al., 2015; Wacker et al., 2017), although there could be cell type dependency due to intrinsic differences between hepatoma cells and HeLa cells used in the different experiments (Schmuckli-Maurer et al., 2017). While this has led to a discussion on whether host autophagy is a friend or foe during liver stage infection (Coppens, 2017), an emerging view is that the liver stage *Plasmodium* development could represent a non-canonical form of autophagy, recently termed *Plasmodium* Associated Autophagic-like Response (PAAR; Coppens, 2017; Wacker et al., 2017; Agop-Nersesian et al., 2018).

**FIGURE 2:**
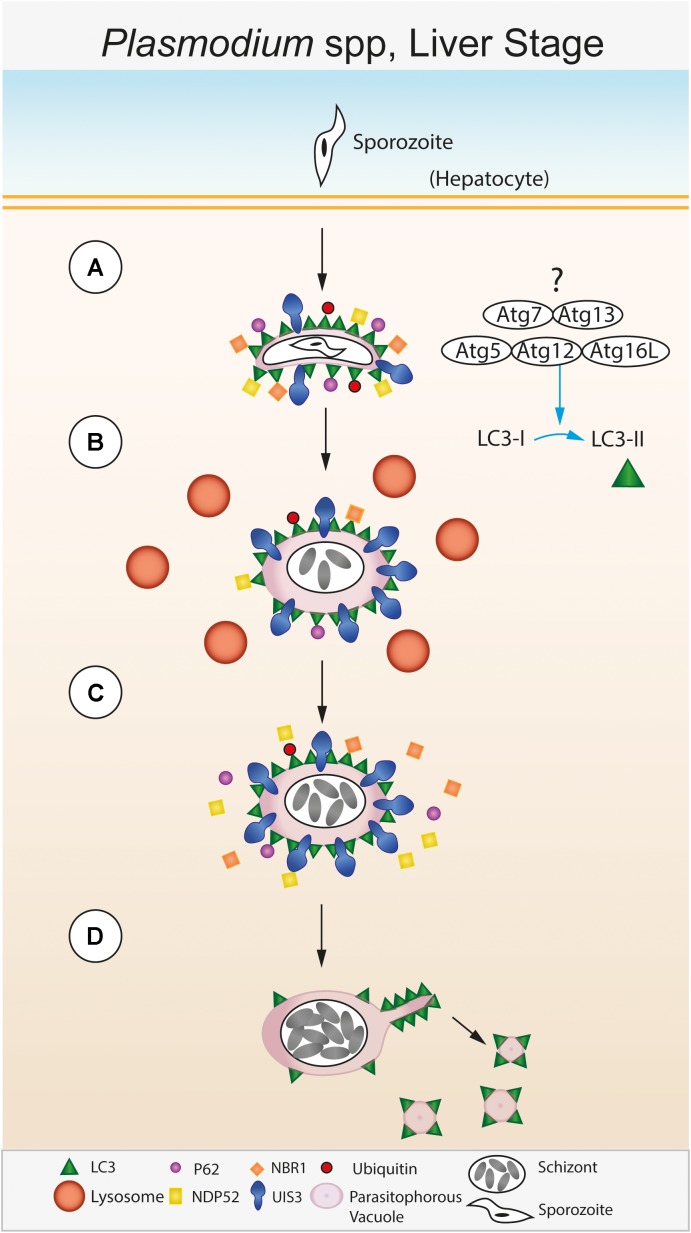
Autophagic control of the liver stage of *Plasmodium* spp. **(A)** The sporozoite stage of the *Plasmodium* parasite invades a hepatocyte within the liver. Following invasion, the parasite resides within a membrane bound parasitophorous vacuole (PV) within the host cell cytosol (Meis et al., 1983; Lingelbach and Joiner, 1998; Nyboer et al., 2017). The PV membrane (PVM) is recognized immediately after infection by the host. Lipidated LC3 is deposited onto the PVM followed by recruitment of host effector proteins including p62, NDP52, NBR1, and ubiquitin (Schmuckli-Maurer et al., 2017). **(B)** The *Plasmodium* PVM resident protein UIS3 sequesters LC3 at the PVM surface and prevents further p62/NDP52/NBR1/ubiquitin binding (Real et al., 2018). Meanwhile, the PVM is surrounded by lysosomes, however, lysosomal fusion and acidification of the PVM compartment does not occur. **(C)** This step is required for parasite development within the host cell and leads to further schizont replication. **(D)** During late stage parasite development, LC3 is shed from the PVM via sequestration by the *Plasmodium* tubo-vesicular network (TVN) and subsequent scission from the PVM (Agop-Nersesian et al., 2017).

The molecular mechanisms of the role of host autophagy during liver stage *Plasmodium* infection are being unraveled (**Figure 2**). A hallmark of *Plasmodium* development in the liver stage is the rapid acquisition of LC3, as well as its binding proteins p62, NBR1, NDP52, along with ubiquitin on the PVM (Schmuckli-Maurer et al., 2017). This suggests that either the parasite is readily recognized by the host or the parasite sequesters and hijacks the host autophagy machinery. There are several striking aspects of the association of LC3 with the PVM that renders it distinct compared to other known forms of autophagy. First, the LC3 decoration on the PVM does not involve the formation of new canonical double membrane autophagosomes, rather LC3 associates with the existing PVM. This is distinct from LAP since sporozoites invade host cells by an active mechanism different from conventional phagocytosis, and the PVM while surrounded by lysosomes does not readily fuse with lysosomes and become acidic, as is the case in LAP. Second, the association of the LC3-binding proteins, including ubiquitin, to the PVM is to a large extent directly mediated by LC3 (Schmuckli-Maurer et al., 2017). This is in contrast to canonical xenophagy, where LC3 recruitment on pathogen vacuole membrane is subsequent to their recognition by receptors. The order of recruitment of LC3 binding proteins to the PVM appears reversed in case of *Plasmodium* liver stage infection, leading to the idea of an “inverted” recruitment of LC3 associated proteins on the PVM (Schmuckli-Maurer et al., 2017). Third, the association of LC3 itself with the PVM is temporary (Prado et al., 2015), with LC3 dissociating from the PVM during the later stages of parasite development. Both recruitment of LC3 onto the PVM at an early time point post-infection (as early as a few minutes) and the disappearance of LC3 from the PVM at later time points (after 40 h) are necessary for proper parasite development (Prado et al., 2015; Agop-Nersesian et al., 2017). Fourth, LC3 recruitment to the PVM is dependent on lipidation of LC3 (Prado et al., 2015), suggesting that the LC3 conjugation machinery involving upstream ATGs such as ATG5 are actively involved in the process. However, initiation complexes of autophagy such as FIP200 are not required (Wacker et al., 2017).

The factors that trigger the LC3 conjugation system upon *Plasmodium* infection and how lipidated LC3 is recruited to the PVM are not clear. However, recent evidence suggests that the parasite protein UIS3 directly binds to and retains LC3 on the PVM (Real et al., 2018). Multiple lines of evidence attest to the role of *Plasmodium* UIS3 in intersecting with the host autophagy machinery by interacting with LC3: first, while *uis3(-)* parasites are arrested in development in wild-type hepatocytes, they develop normally in ATG5^-/-^ MEFs, arguing strongly for a central role for UIS3 in interaction with host autophagy. Second, exogenously expressed UIS3 interacts with LC3 in HeLa cells, which is confirmed by direct *in vitro* interaction of purified recombinant LC3 and UIS3. Third, by modeling the LC3-UIS3 interaction interface, critical residues were identified on UIS3 that were important for binding to LC3. Mutant UIS3 where these residues, singly or in combination, are mutated to alanine, do not show binding to LC3. Interestingly, the residues on UIS3 do not conform to a conventional LIR motif, suggesting a non-canonical interaction (Real et al., 2018). An emerging view is that UIS3, by sequestering LC3 onto the PVM, blocks LC3 binding to its other target proteins, resulting in an inhibitory effect on the host autophagy machinery. Evidence for this comes from the reduced p62 degradation observed in UIS3 transfected HeLa cells (Real et al., 2018). However, since LC3 interacting proteins such as p62, NDP52 also bind to the PVM by binding to LC3, it might be possible that the UIS3 mediated inhibitory effect is either incomplete or occurs after the first wave of LC3 targeting and its associated proteins have already bound to the PVM. The specificity and non-canonical nature of LC3-UIS3 interaction and the essentiality of UIS3 for parasite development raises the exciting possibility of exploring small molecule disruptors of this protein-protein interaction to target liver stage development. Direct structural information on the UIS3-LC3 interface will be crucial for such studies.

LC3 dissociating from the PVM is necessary for late stage parasite development (Prado et al., 2015; Agop-Nersesian et al., 2017). An interesting “exit” mechanism has been proposed by Heussler and colleagues using elegant live cell imaging experiments, wherein the tubo-vesicular network (TVN) surrounding the PVM siphons off LC3 from the expanding PVM and sheds it into the host cell cytoplasm as vesicles (Agop-Nersesian et al., 2017). This interesting observation raises several exciting questions. What is the fate of the interacting UIS3 during this step? How is the flow of membrane from PVM to TVN regulated while the PVM itself is actively expanding? What are the roles of the host cytoskeletal elements, including the acto-myosin complex, if any, in this process? What mechanisms ensure and regulate sufficient forces and membrane tension for such a sequestration effect? What are the mechanisms involved in scission of the vesicles from TVN, what prevents the “backflow” of LC3 from TVN to PVM? What prevents the re-recruitment of LC3 to PVM? These ongoing studies from multiple laboratories have thus opened up several new and exciting lines of enquiry (**Box 1**).

**Box 1. A collection of unanswered questions concerning each pathogen discussed in this review.**

***Plasmodium* Spp. Liver Stage**

• What are the triggers of the autophagy nucleation machinery and LC3 lipidation upon *Plasmodium* infection?
• Why is dissociation of LC3 required for late stage parasite development? Is it coupled to other processes happening at that time, such as merosome formation?
• What role does host autophagy play in human malarial infections? Does it have a role in hypnozoite biology of *P. vivax*?

***Toxoplasma gondii***

• Why is autophagic control of *Toxoplasma* seemingly different in murine and human cells?
• Do all human cells control *Toxoplasma* via the same autophagic mechanism?
• How do Atg proteins control the recruitment of IRGs and GBPs to the parasite vacuole in murine cells?

***Leishmania* Spp.**

• What is the mechanism of nutrient acquisition by *Leishmania* via autophagy?
• How does autophagy lead to *Leishmania*-specific T cell attenuation?

**Fungal Spp. (*Cryptococcus neoformans, Candida albicans* and *Aspergillus fumigatus*)**

• What are the defining characteristics of LC3-associated phagocytosis (LAP) aside from LC3 recruitment to the phagosome?
• Does LC3 recruitment to the phagosome during fungal infection always induce LAP?
• Why do some fungal pathogens appear to be more susceptible to LAP than others?
  ◦ Do fungal pathogen induce species-dependent variations of LAP within host cells or have the pathogens evolved to subvert LAP in different ways?
• What is the opsonic receptor responsible for LC3 recruitment to *C. neoformans*?
  ◦ Is Syk activation/reactive oxygen species generations still required to induce LAP following opsonic phagocytosis?

Most of the results listed above come from studies using murine malarial parasites, notably *P. berghei*. It will be important to address the relevance of these findings during the liver stage infections of human malarial parasites, *P. falciparum* and *P. vivax*. Boonhok et al assessed the effect of IFNγ treatment in hepatocytes during *P. vivax* infections (Boonhok et al., 2016), and identified a LAP-like process that kills *P. vivax* upon stimulation with IFNγ. This process involves autophagy nucleation factors like ATG5, Beclin1, but not the initiation factor ULK1 (Boonhok et al., 2016), consistent with the observations from *P. berghei*. While this study highlights the involvement of IFNγ in *P. vivax* liver stage development, the role of basal autophagy, autophagy components and PAAR like response remain to be elucidated. Given the unique preference of *P. vivax* for dormancy during its liver stages development (Markus, 1980; Krotoski et al., 1982), it is particularly tempting to speculate if there could be differential recruitment of selective host autophagy components between the actively growing schizont and the dormant hypnozoite forms.

Exciting new concepts have emerged in the recent past on the interaction of *Plasmodium* spp with host cell autophagy machinery during the liver stages. Several unusual features define this interaction. They include the necessary, but transient recruitment of LC3 and its binding proteins to the PVM, the “inverted” nature of this recruitment, with LC3 binding to PVM preceding that of its binding proteins, the inhibitory effect of a parasite protein UIS3 on host autophagy machinery via its non-canonical interaction with LC3 and the interesting “exit” mechanism of LC3 from PVM. These observations have opened up new avenues in this rapidly expanding area of research. The relevance of these concepts to human malarial parasites *P. falciparum* and *P. vivax*, and the potential of this interaction for drug discovery make this a particularly exciting if challenging area for future research.

## Autophagy and *Toxoplasma gondii*

*Toxoplasma gondii*, like *Plasmodium*, is an apicomplexan parasite that leads a ubiquitous intracellular life. The sexual stage of *Toxoplasma’s* life cycle is confined to the feline, while the asexual stage is promiscuously found in all warm-blooded animals. Due to this trait and the fact that *Toxoplasma* establishes a chronic infection in brain and muscle tissue, it can arguably be considered the most successful parasite on the planet with human infection rates of 30% (Torgerson and Mastroiacovo, 2013). *Toxoplasma* infection in immunocompetent people is mostly asymptomatic but can lead to ocular disease when infected with certain parasite strains. Immunocompromised individuals and neonates are also at risk of severe health problems and death (Hill and Dubey, 2002). In North America and Europe, *Toxoplasma* is mostly present as one of three classical strains, types I, II, and III, while an expansion of strain diversity has occurred in South America (Ajzenberg et al., 2004).

### CD40-Induced Autophagic Host Control of *Toxoplasma gondii*

Autophagic control of *T. gondii* requires stimulation of the host cells. Almost 20 years ago, it was found that CD40 ligand deficient mice are unable to control *in vivo* replication of *Toxoplasma* in the brain (Reichmann et al., 2000). CD40 activation also controls parasite growth in peripheral tissues during the acute phase of infection (Subauste and Wessendarp, 2006), as well as cerebral and ocular toxoplasmosis (Portillo et al., 2010). However, the predominance of its role, alongside the IFNγ-induced autophagic pathway (see below), in controlling *Toxoplasma* in murine macrophages *ex vivo* has been questioned (Zhao et al., 2007). CD40 ligation was recognized to induce the autophagic clearance of the parasite (Andrade et al., 2006). To date, this mechanism is mostly studied in murine macrophages and probably exists in non-hematopoietic murine cells (Van Grol et al., 2013) and in human macrophages (Andrade et al., 2006).

**FIGURE 3:**
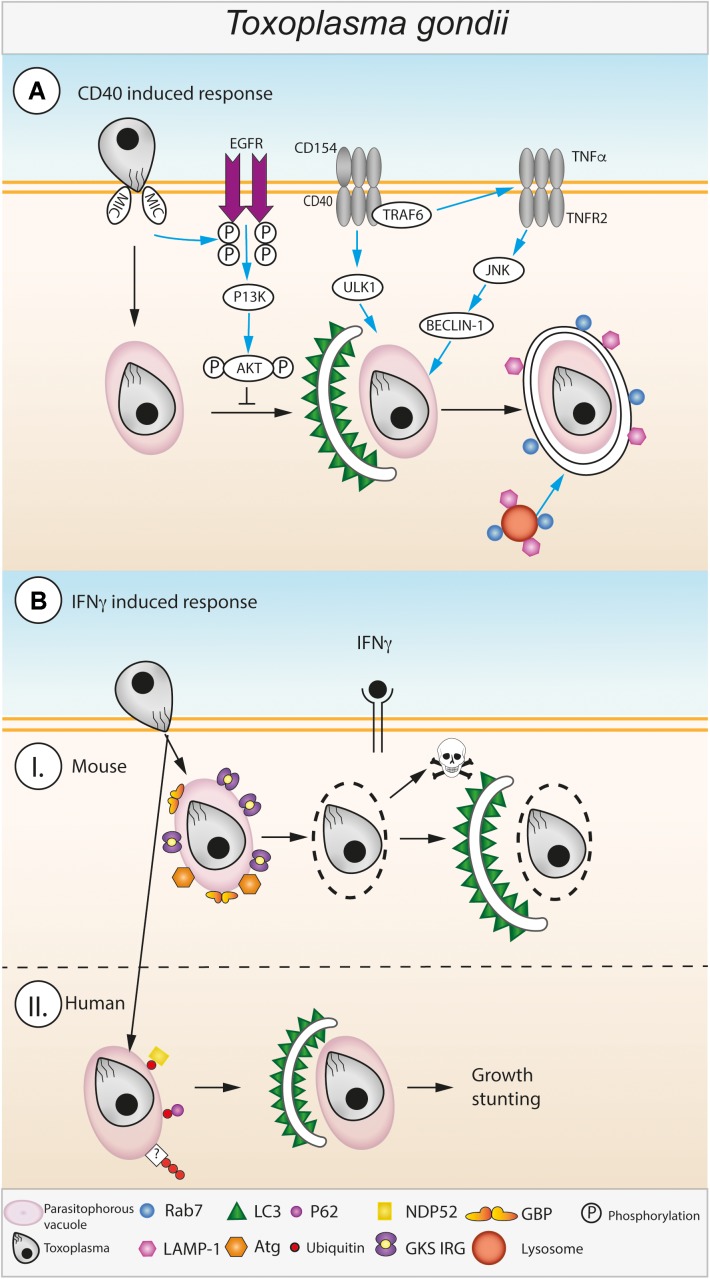
Autophagic control of *Toxoplasma gondii*. **(A)** CD40 induced autophagy during *Toxoplasma* infection. *Toxoplasma* enters into host macrophages via an active invasion process, the parasite resides in the host cytosol within a parasitophorous vacuole (PV). Host autophagy pathways are induced against *Toxoplasma* via interactions between CD40 expressed on the cell surface of infected macrophages and CD4^+^ T cells expressing CD154 (Andrade et al., 2006). CD40/CD154 ligation leads to recruitment of TRAF6 to CD40 which triggers increased TNFα secretion (Mukundan et al., 2005; Subauste et al., 2007). CD40 mediated ULK1 activation and TNFR2 mediated JNK/Beclin-1 activation leads to the formation of a double membraned autophagosome around the *Toxoplasma* PV (Andrade et al., 2005). The host’s autophagic response is actively inhibited by the *Toxoplasma* derived protein MIC which activates host EGFR which in turn activates PI3K leading to activation of the autophagy suppressor protein AKT (Muniz-Feliciano et al., 2013). *Toxoplasma* is destroyed by recruitment of Rab7 and LAMP1 and the subsequent fusion the PV with lysosomes (Andrade et al., 2006). The pathway depicted to the left of the dashed line is found in unstimulated and CD40-stimulated cells. **(B)** IFNγ induced autophagy during *Toxoplasma* infection. **(I)** In mouse cells stimulated with IFNγ the *Toxoplasma* PVM is disrupted by recruitment of GKS-motif containing Immunity Related GTPases (GKS IRG) and Guanylate Binding Proteins (GBPs) to the outer surface of the PVM (Degrandi et al., 2007; Virreira Winter et al., 2011; Yamamoto et al., 2012; Selleck et al., 2013). Disruption of the PV leaves the parasite exposed to attack by host autophagy pathways, characteristic autophagosomes (double membrane, LC3 decorated) form leading to destruction and digestion of the parasite (Ling et al., 2006). **(II)** In human cells, the mechanisms responsible for IFNγ mediated destruction of *Toxoplasma* via autophagy are less well-known. Instead of disrupting the PVM, human cells target it with ubiquitination which leads to the subsequent recruitment of ubiquitin binding proteins, e.g., p62 and NDP52. Recruitment of p62 and NDP52 leads to autophagosome formation around the PV via an unknown process which leads to restriction of parasite growth within the cell (Selleck et al., 2015; Clough et al., 2016).

Presumably following a canonical autophagy route, upon CD40 ligation, LC3 localizes around the *Toxoplasma* parasitophorous vacuole (PV) within 6 h as well as the late endo-lysosomal markers LAMP1 and Rab7 (**Figure 3A**). This suggests that CD40 ligation directs the PV to fuse with endo-lysosomal compartments (Andrade et al., 2006). Importantly, the PVM seems to stays intact throughout this process, however, more detailed investigation is required to confirm this hypothesis. CD40 ligation to combat *Toxoplasma* requires synergy with TNFα (Andrade et al., 2005). CD40 recruits TRAF6 to an intracellular binding site serving two purposes: to enhance autocrine production of TNFα (Mukundan et al., 2005) and to engage TRAF6 signaling downstream of CD40 by synergizing with TNFα to activate autophagy (Subauste et al., 2007) (**Figure 3A**). Resultingly, Beclin1 and ULK1 synergistically signal to promote autophagic clearance of *Toxoplasma* (Liu et al., 2016).

*Toxoplasma* has to maintain the non-fusogenic nature of the PV to ensure tachyzoite survival. The following proposed mechanism was studied in many cell types including human brain endothelial cells, retinal cells, as well as mouse endothelial cells, microglial cells and macrophages (Muniz-Feliciano et al., 2013). *Toxoplasma* type I and II activate EGFR-Akt signaling in host cells, preventing the targeting of the PVM by the autophagy protein LC3 and thus avoiding Beclin1- and Atg7-dependent autophagic clearance (Muniz-Feliciano et al., 2013) (**Figure 3A**). Phosphorylation of Akt increases with live parasite infection in an IFNγ-independent manner. Two parasite microneme (MIC) proteins containing EGF domains, MIC3, and MIC6, are important contributors to this process (Muniz-Feliciano et al., 2013). Another study recently proposed that in a second mechanism also active in non-CD40 activated cells, *Toxoplasma* invasion activates a focal adhesion kinase (FAK)-Src-EGFR transactivation to STAT3 pathway, which inhibits autophagosome formation and thus *Toxoplasma* killing (Portillo et al., 2017). In line with these findings, another study reported that Gefitinib, an EGFR inhibitor, decreased parasite replication in HeLa cells (Yang et al., 2014).

A common theme and sometimes prerequisite in autophagic control of intracellular, vacuolated pathogens is the exposure of the pathogen to the cytoplasm. This can either happen spontaneously, such as for *Salmonella typhimurium*, or be driven by host defense proteins, for example for *Chlamydia* and *T. gondii*. Gamma interferon is central to upregulating the expression of host GTPases, the Immunity Related GTPases (IRGs) and Guanylate Binding Proteins (GBPs), both responsible for disrupting pathogen vacuoles by a yet undetermined mechanism (Degrandi et al., 2007; Virreira Winter et al., 2011; Yamamoto et al., 2012; Selleck et al., 2013). *Toxoplasma* then dies in the cytoplasm and is potentially cleared by canonical host cell autophagy, a striking ultrastructural observation now made well over 10 years ago (Ling et al., 2006) (**Figure 3B**). Here, a dependence on the IRG Irgm3 was observed, which localizes to the autophagosomal membranes enveloping the naked parasite (Ling et al., 2006). Another report at that time found LC3 in close vicinity to the PV, suggesting a similar role for autophagy in tachyzoite elimination (Martens et al., 2005).

A hint that the story would not be straightforward arrived with the observation that Atg5 restricted *Toxoplasma* in murine macrophages, but that the PVs were not uniformly acidic in the form of LAMP1 positivity (Zhao et al., 2008). It is now clear that autophagy proteins including the E3-like autophagy complex localize to and recruit host IRGs and mGBPs to the PVM (Zhao et al., 2008; Khaminets et al., 2010; Choi et al., 2014; Park et al., 2016) (**Figure 3B**). For example, Atg5 is essential for the recruitment of Irga6 and Irgb6 to the PV in mouse macrophages, fibroblasts and granulocytes (Zhao et al., 2008; Khaminets et al., 2010). In the absence of Atg5, Irga6, Irgb6 and Irgd aggregated in the host cytoplasm (Zhao et al., 2008; Khaminets et al., 2010). Irgb6 and mGBPs are recruited to the PV in dependence of Atg7 and Atg16L1, yet with Atg9a and Atg14 being dispensable (Ohshima et al., 2014). Similarly, Atg3 is necessary for loading of IRGs and mGBP2 (and possibly other GBPs) onto the PVM and control of *Toxoplasma* infection (Choi et al., 2014; Haldar et al., 2014). Even though the mechanism is unclear, these Atg proteins appear to activate the GTPases, as it was found that a GTP-locked, constitutively active, IRG protein mutant could overcome the targeting defect in Atg3 and Atg5 deficient cells (Haldar et al., 2014). Equally, depletion of all LC3 homologs including GABARAP, GABARAPL1, and GABARAPL2 (GATE-16), led to decreased targeting of the IRGs to the PVM (Park et al., 2016). Relocating the Atg12-Atg5-Atg16L1 complex that marks the LC3 conjugation site onto alternate target membranes led to the host GTPases accumulating at the new target membranes rather than the PVM (Park et al., 2016).

In terms of direct localization of Atg proteins, the Atg12-Atg5-Atg16L1 complex has been postulated to target to the PVM using effector proteins that link phosphoinositides to the Atg complex (Park et al., 2016) (**Figure 3B**). Alternatively, the PVM may be recognized by “missing self,” similarly described for GMS IRGs (Haldar et al., 2013; Maric-Biresev et al., 2016; Park et al., 2016). Regardless, currently the factors governing the initial recruitment of Atg proteins to the PVM are unclear. In summary, it is clear, however, that this early involvement of Atgs does not lead to canonical autophagy, since the Atg proteins do not promote the formation of an isolation membrane at the PVM prior to PV breakage (Martens et al., 2005; Ling et al., 2006). Autophagy Atg proteins thus serve a non-canonical autophagy function in *Toxoplasma* control in their capacity to promote recruitment of host GTPases to the PVM. After PVM destruction, the observation of autophagic membranes around the exposed parasite implies their participation in a classical autophagic role or alternatively a LAP-like clearance of the *Toxoplasma* PV.

Interestingly, in humans, no role for the IFN-stimulated IRGs in *Toxoplasma* control has been documented and thus far, the PVM has never been observed as disrupted. This is possibly a consequence of the human genome containing only two IRGs, both non-interferon inducible, IRGC, which is testis specific and IRGM (Bekpen et al., 2005). Humans do possess 7 IFNγ-inducible GBPs. Human GBP1-5 and hGBP1 recruit to *Toxoplasma* in HAP1 and mesenchymal stromal cells, respectively (Ohshima et al., 2014; Qin et al., 2017). However, no recruitment of hGBP1 to the *Toxoplasma* PVM was found in A549 cells (Johnston et al., 2016). Thus, either absence of IRG protein targeting to the *Toxoplasma* PV protects its rupture, or the cell type or circumstance where this may happen has not been found.

Autophagy proteins do play a role in *Toxoplasma* infection of the human epithelial HeLa cell line (Selleck et al., 2015; Clough et al., 2016) (**Figure 3B**). Ablating Atg16L1 and Atg7 resulted in increased parasite replication. This again was described as non-canonical autophagy, as it did not lead to lysosomal fusion, with no evidence for LAMP1 staining. Instead, parasites were growth-restricted by an unknown mechanism involving recruited LC3B and membranes to the type II and III PV (Selleck et al., 2015) (**Figure 3B**). Other studies demonstrated that the key autophagy mediators Atg5 and Atg16L1are not required for parasite restriction in human foreskin fibroblast (HFF) and HAP1 cells, respectively (Niedelman et al., 2013; Ohshima et al., 2014). Again, this may be a cell-type specific difference in human *Toxoplasma* restriction. A common theme between human epithelial and endothelial cell types seems to be ubiquitin recognition of type II and III PVs (Selleck et al., 2015; Clough et al., 2016). Ubiquitin recognition is the prerequisite to parasite destruction, a process that involves the autophagy adaptor proteins p62 and NDP52, but again, no obvious PVM disruption (Selleck et al., 2015; Clough et al., 2016). Interestingly, minimal recognition by galectin 8 was found in an IFNγ and type II parasite specific manner, potentially indicating a slight permeability of the PVM (Clough et al., 2016).

Much progress has been made to elucidate how autophagy can restrict *Toxoplasma* in murine cells, with some understanding how this pathway operates in human cells. It has become clear that there are differences in pathways depending on organism infected, cell type under study and *Toxoplasma* strain. It will be critical to unravel these differences, as well as understand their importance during human infection (**Box 1**). For example, CD40 ligation has been suggested to restore IFNγ and IL12 production *ex vivo* in patients with hyper IgM syndrome, possibly linking some of the discussed pathways (Subauste et al., 1999). Conversely, studies have also pointed out that *Toxoplasma* can benefit from autophagic degradation as a means to provide nutrients (Wang et al., 2009; Pernas et al., 2018). Further studies will be needed to address how the autophagy-*Toxoplasma* interplay is balanced.

## Autophagy and *Leishmania*

*Leishmania* spp. are protozoan parasites that cause a variety of diseases in humans ranging from cutaneous lesions to visceral leishmaniasis. *Leishmania* is ranked second in mortality to malaria among parasitic infections and is primarily found in tropical and subtropical countries (GBD 2015 DALYs and HALE Collaborators, 2016). *Leishmania* invades macrophages in the dermis (Liu and Uzonna, 2012). The promastigote stage at that point evolves into the amastigote stage in the phagolysosome. Thus, *Leishmania* has developed ways to block phagolysosomal maturation in order to survive (Kaye and Scott, 2011). Amastigotes multiply and disseminate to the reticulo-endothelial system through the lymphatic system, then infiltrating macrophages in the bone marrow. Autophagy could thus benefit the parasite by providing nutrients or play a role in pathogen defense.

Several *Leishmania* species have been found to induce autophagy. This is thought to be a means for the parasite to acquire critical nutrients. *Leishmania infantum* disease severity seems to be associated with upregulation of the autophagy genes Atg7 and LC3, as well as the LAP-like accumulation of LC3 around the parasite vacuoles (Esch et al., 2015). Increased *Leishmania amazonensis* parasite burden could be found in Balb/c mice after the induction of autophagy (Pinheiro et al., 2009) and the parasite has itself been found to induce autophagy in macrophages, concurrent with an increased infection index after inhibiting autophagy with 3-methyladenine (Cyrino et al., 2012). Patient data from a *Leishmania donovani-*infected individual showed induction of autophagy by LC3 conversion from the patient’s bone marrow samples (Mitroulis et al., 2009). Direct acquisition of macromolecules has been demonstrated for *Leishmania mexicana* via an autophagy-sensitive pathway (Schaible et al., 1999).

Induction of autophagy by *Leishmania* can be a means to attenuate T cell responses against the parasite. Single bilayers positive of LC3 seem to surround apoptotic *Leishmania major* with the consequence of dampening the parasite-directed CD4 T cell response (Crauwels et al., 2015). Reducing T cell exhaustion by blocking PD1-L signaling inhibited autophagy and reduced *Leishmania donovani* burden (Habib et al., 2018).

Only one report has seemingly observed autophagy as a mechanism for *Leishmania* destruction. *L. major* was found to increase the presence of autophagosomes, vacuoles and myelin-like structures, concurrent with the clearance of amastigotes (Frank et al., 2015). Clearly more mechanistic work is needed to elucidate the exact model of interaction between *Leishmania* species and the host autophagy machinery (**Box 1**).

## Autophagy and Fungal Pathogens

Invasive fungal infections cause around 1.5 million deaths per year, the majority of deaths are due to just three fungal species –*Cryptococcus neoformans*, *Candida albicans*, and *Aspergillus fumigatus* (Brown et al., 2012). All three species pose a significant risk to individuals who have become immunocompromised, e.g., via HIV AIDS, hematological malignancies, major physical trauma or immune suppression therapy for solid organ transplant. *C. neoformans* and *A. fumigatus* are environmental fungi that can cause respiratory infection following inhalation of infectious spores, *A. fumigatus* remains within the lungs where it causes severe inflammation and tissue damage (van de Veerdonk et al., 2017), whereas *C. neoformans* disseminates to the central nervous system where it can cause fungal meningitis (Ma and May, 2009; Evans and May, 2014; Gibson and Johnston, 2015). *C. albicans* is a commensal organism that can opportunistically outgrow its niche in the intestinal tract, oral cavity or vaginal cavity if an individual is immunocompromised. Fatal candidiasis occurs when *C. albicans* invades epithelial barriers and enters the bloodstream resulting in sepsis (Mayer et al., 2013). Initially, *C. neoformans* resists the intracellular killing within the macrophage and is able to proliferate within mature phagosomes. Infected macrophages require a CD4^+^ Th1 helper cell mediated adaptive immune response to control intracellular infection (Kawakami et al., 1995; Voelz et al., 2009). Like *C. neoformans*, *C. albicans* is able to survive within the macrophage phagosome, however, *C. albicans* is able to form hyphae, which disrupt host cell membranes leading to the escape of the fungus (Mayer et al., 2013). *A. fumigatus* spores or conidia are inhaled into the alveolar space, where alveolar macrophages initially phagocytose and kill conidia (van de Veerdonk et al., 2017).

### LC3-Associated Phagocytosis (LAP)

**FIGURE 4:**
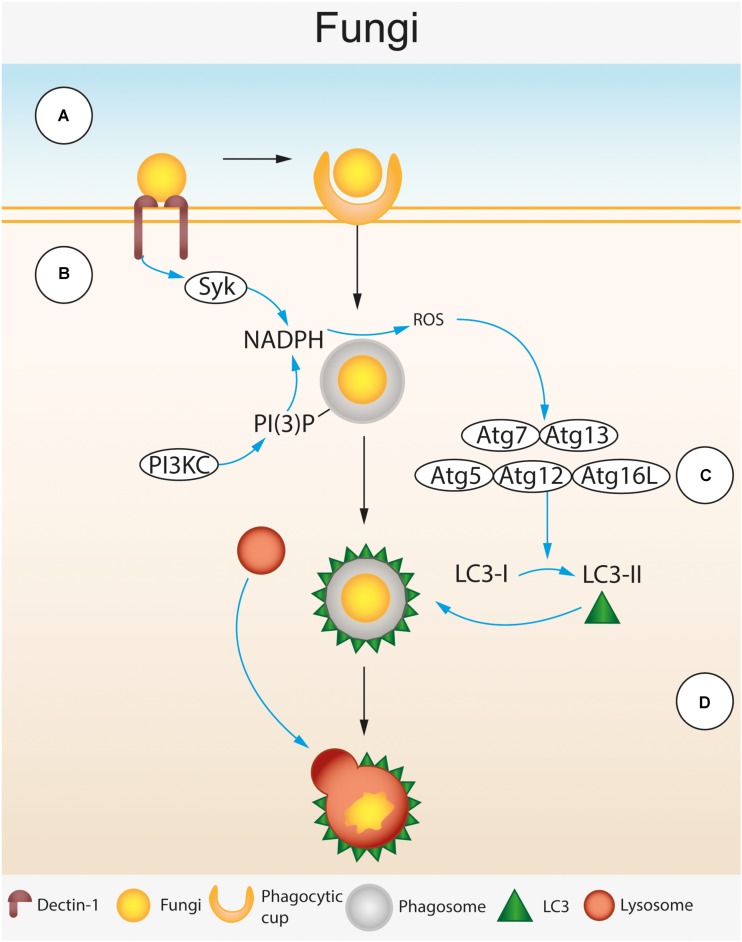
Autophagic control of Fungal spp. LC3-associated phagocytosis (LAP) during fungal infection of macrophages. **(A)** β 1,3 glucan residues in the fungal cell wall are recognized by the cell surface receptor Dectin-1 expressed on the surface of the macrophage (Brown and Gordon, 2001; Brown et al., 2002; Brown, 2006). Dectin-1 recognition leads to phagocytosis of fungal cells. Following phagocytosis, the phagocytosed fungus is enclosed by a single membraned phagosome within the cytosol. **(B)** Dectin-1 activation triggers spleen tyrosine kinase (SyK) activation (Gantner et al., 2003). Activated Syk and phosphatidylinositol 3-phosphate (PI(3)P) deposited on the surface of the phagosome by the phosphoinositide 3-kinase complex (PI3KC) recruit NADPH oxidase leading to the production of reactive oxygen species (ROS) within the phagosome (Gantner et al., 2003). **(C)** ROS production attracts the LC3 lipidation complexes (Atg7-Atg13 and Atg5-Atg12-Atg16L) that convert LC3-I to LC3-II and deposit it on the phagosome surface (Ma et al., 2012; Kyrmizi et al., 2013). **(D)** LC3 deposited on the phagosome membrane leads to lysosomal fusion, acidification of the phagosome and destruction of the fungus.

*Cryptococcus neoformans, C. albicans*, and *A. fumigatus* are targeted by host autophagy proteins during infection and remain within single membrane phagosomes throughout. As previously discussed LAP requires some, but not all of the canonical autophagy machinery. LAP is triggered by host cell pattern recognition receptors (PRRs) that recognize pathogen-associated molecular patterns (PAMPs) unique to the pathogen. PI(3)P is deposited on the phagosome membrane by the PI3KC3/Rubicon/UVRAG complex, this recruits NADPH oxidase and NOX2 to the phagosome resulting in production of reactive oxygen species (ROS) which attracts the LC3 conjugation complexes Atg7-Atg3 and Atg5-Atg12-Atg16L, LC3 as well as Atg3 and Atg4. The end result of LAP is the lipidation of LC3-I into LC3-II, which is attached to the phagosome membrane to form a structure called the LAPosome. LAPosomes are able to fuse with lysosomes leading to phagosome maturation and destruction of the pathogen (**Figure 4**). Following destruction, the pathogen is digested, it is then possible for components of the pathogen to be passed to endosomal PRRs such as TLR2 and TLR7 or processed for antigens that can be presented on MHC-II complexes for antigen presentation.

#### Recognition of Fungal Pathogens During LAP

LC3-associated phagocytosis mediated deposition of LC3 onto phagosome membranes can be induced by toll-like receptor (TLR) activation, but in the context of fungal infection the C type lectin receptor Dectin-1 can also mediate LAP. Dectin-1 is a cell surface PRR expressed mainly on myeloid cells that recognizes β-1,3-glucan – a polysaccharide found in the fungal cell wall (Brown and Gordon, 2001; Brown et al., 2002; Brown, 2006). Genetic mutations in Dectin-1 are known to increase susceptibility to *C. albicans* and *A. fumigatus* (Marakalala et al., 2011). Ligand binding to Dectin-1 leads to phosphorylation of an ITAM located on the cytoplasmic tail of the receptor. This subsequently recruits and activates spleen tyrosine kinase (SyK) which activates NADPH oxidase leading to production of ROS in the phagosome (Gantner et al., 2003) (**Figure 4**). Dectin-1 activation is required for LC3 recruitment to phagosomes containing *C. albicans* and *A. fumigatus* within infected macrophages (Ma et al., 2012; Kyrmizi et al., 2013). Dectin-1 mediated LC3 recruitment is Syk dependent and relies on ROS generation by NADPH oxidase (Ma et al., 2012; Kyrmizi et al., 2013).

The PPR that leads to LC3 recruitment to *C. neoformans* phagosomes is still not known. Nicola et al. report that only antibody-opsonized *C. neoformans* recruit LC3 (Nicola et al., 2012), however, LC3 recruitment to phagosomes containing unopsonized *C. neoformans* cells has been reported by Qin et al. (2011). This suggests that Dectin-1 activation may not be fully responsible for mediating LC3 recruitment to phagosomes containing *C. neoformans*. Unopsonised *C. neoformans* cells are very poorly phagocytosed by host macrophages (Evans et al., 2015; Bojarczuk et al., 2016; Lim et al., 2018), due to the polysaccharide capsule produced by *C. neoformans* during infection that can hide β-1,3-glucan from Dectin-1. It is possible that a host recognition receptor other than Dectin-1 is responsible for LAP induction against *C. neoformans*, in this respect Fc-receptor activation has been shown to induce LC3 recruitment to phagosomes (Huang et al., 2009). Interestingly, recent research by Lim et al. shows that although unopsonised *Cryptococcus* cells are poorly phagocytosed by macrophages, the phagocytosis that does occur is Syk-dependent and can be blocked with the Syk inhibitor piceatannol. Furthermore, Syk activation was found to localize to an area around phagocytic cup formation during phagocytosis and the uptake of non-opsonized *Cryptococcus* cells could be blocked by pharmacological or genetic ablation of Dectin-1 (Lim et al., 2018). This suggests that Dectin-1 activation is seen during macrophage recognition of *C. neoformans*, but further work must be performed in order to explore whether this leads to LC3 recruitment.

#### Recruitment of LC3 to Phagosomes Containing Fungi

One of the defining features of LAP is the deposition of LC3 on the phagosome membrane. LC3 recruitment to phagosomes containing *C. neoformans* infection has been observed as early as 1 h post-infection and persists for at least 24 h post infection. Recruitment levels differ between studies but range from ∼40 to 80% at 12 h post-infection (Qin et al., 2011; Nicola et al., 2012), furthermore, as discussed above, Nicola et al. show that for *C. neoformans* the route of uptake can determine LC3 recruitment. Phagosomes containing both unopsonized (Qin et al., 2011; Nicola et al., 2012) and opsonized (Pandey et al., 2017) *C. neoformans* cells recruit LC3. It has been found that phagocytosis of *C. neoformans* cells by macrophages leads to the activation of the host autophagy initiation complex (AIC) as well as upstream regulatory components LKB1 and AMPKα, which regulate autophagy induction through their kinase activity. Depletion of AIC components (ULK1, Atg13, and FIP200) and AMPKα reduces LC3 recruitment to *C. neoformans* containing phagosomes (Pandey et al., 2017). On phagosomes containing *C. albicans*, LC3 recruitment is observed for both live and heat-killed cells, heat-killed *Candida* elicit higher LC3 recruitment compared to live at 30 min post infection, however, at 60 min this phenotype is reversed (Tam et al., 2014). This could suggest that *C. albicans* actively inhibits LAP, but it is also possible that the heat killing leads to increased availability of LAP activating PRRs by changing the cell wall composition. Recruitment of LC3 to phagosomes containing *C. albicans* is Dectin-1/ROS dependent and leads to increased intracellular killing of *C. albicans* by macrophages (Tam et al., 2014). For *A. fumigatus*, Kyrmizi et al. report that monocyte phagosomes containing the *Aspergillus* conidia only recruit LC3 after the conidia begin to germinate or “swell” within the phagosome. The swelling process leads to changes in the cell wall composition of conidia including increased β-1,3-glucan display. As with *C. albicans*, LC3 recruitment to *A. fumigatus* conidia was ROS dependent. Furthermore, monocytes from patients with Chronic Granulomatous Disease (CGD), who have inactivating mutations in NADPH oxidase, fail to recruit LC3 to swollen conidia (Kyrmizi et al., 2013).

#### The Contribution of LAP to Host Defense

Although LC3 recruitment to the phagosome has been observed for all three fungi it is still unclear what downstream effects LAP has on fungal infection. A number of studies have investigated genetic knockdown of autophagy related proteins such as Atg5, Atg9a, Atg7, Atg12, and LC3 (Qin et al., 2011; Nicola et al., 2012; Smeekens et al., 2014; Kanayama et al., 2015). For *C. neoformans*, Qin et al. report that Atg5 and Atg9a recruit to infected phagosomes, however, knockdown of these proteins reduced the growth of *C. neoformans* within infected macrophages (Qin et al., 2011), similar findings in respect to Atg5 knockdown are reported by Nicola et al (Nicola et al., 2012). Further evidence that induction of host autophagy promotes *C. neoformans* growth is provided by Pandey et al. who find that knockdown of AIC components leads to reduced growth of the fungus within macrophages (Pandey et al., 2017). Studies in *C. albicans* have revealed conflicting data. Nicola et al. have shown that Atg5-deficient mice are more susceptible to *Candida* infection than wildtype mice and that Atg5 knockdown in J774 murine macrophages decreases LC3 recruitment to phagosomes (Nicola et al., 2012). Additionally, Kanayama et al. have shown that mice with myeloid specific deficiencies in Atg7 are also more susceptible to *Candida* infection (Kanayama et al., 2015). In contrast to this study, Smeekens et al. report that myeloid specific Atg7 knockout does not affect *Candida* susceptibility in mice. Furthermore, a clinical study by Rosentul et al. that analyzed a cohort of patients with SNPs in the *ATG16L* gene found no correlation between SNPs *ATG16L* and susceptibility to oropharyngeal candidiasis (Rosentul et al., 2014). For *A. fumigatus*, Kyrmizi show that Atg5 knockdown in human THP1 macrophages reduces their ability to kill *A. fumigatus.* This phenotype correlated with reduced acidification of phagosomes containing *A. fumigatus* in Atg5^-/-^ cells (Kyrmizi et al., 2013).

It is clear that LAP is induced against intracellular fungal pathogens, however, there are still many unanswered questions (**Box 1**). At a fundamental level, a better understanding is required about what constitutes LAP. LC3 recruitment to the phagosome is currently one of the only hallmarks to define LAP. As discussed above, studies investigating genetic ablation of autophagy-related genes remain inconclusive as to whether LC3 recruitment to the phagosome leads to improved host defense. The link between LAP and host defense appears to be strongest for *A. fumigatus* (Kyrmizi et al., 2013), while data for *C. albicans* is currently inconclusive (Nicola et al., 2012; Rosentul et al., 2014; Smeekens et al., 2014; Kanayama and Shinohara, 2016) and phagosomes containing *C. neoformans* recruit LC3 but autophagy appears to be required for fungal growth in the phagosome (Qin et al., 2011; Nicola et al., 2012). Interpreting these studies to produce a gestalt picture of LAP’s importance in the defense against fungal pathogens is difficult not only because of the diversity of these fungi, but also because of the variety of strains and models used in each study. One standout issue is that the genes targeted by these studies are also involved in canonical autophagy and therefore their knockdown may affect other processes within the host. It is necessary at the moment to target these genes because very few LAP specific proteins are known other than Rubicon. However, resolving these two pathways should become easier as more components become known. Additionally, very little is also known about what happens to the LAPosome downstream of LC3 recruitment other than its eventual fusion with the lysosome. It is conceivable that *C. neoformans, C, albicans* and *A. fumigatus* could provoke very different host autophagic responses downstream of LAPosome formation which could explain why the outcome for each pathogen is so different. Hopefully as a better understanding of the LAP pathway is gained these questions will be addressed and LC3 recruitment to the phagosome may be seen as more of a staging post to a variety of different pathogen and host dependent outcomes rather than a single fixed pathway.

## Conclusion and Outlook

This review summarizes current knowledge and emerging concepts in the interaction of host cell autophagy with several key eukaryotic pathogens, a field that is only recently emerging, in contrast to bacterial pathogens where autophagy has been established as a crucial mediator in both host defense and bacterial exploitation strategies. While clearly much work needs to be done in the contexts of the individual pathogens addressed, one emergent idea points to the unconventional nature of these interactions, with LAP, or LAP-like processes utilizing selective subsets of core autophagy components playing an important role. The diverse nature of responses and outcomes to LAP-like processes from individual pathogens suggests distinct variations of a core theme, the molecular details of which are likely to emerge in the near future. Importantly, the non-canonical nature of these interactions makes them attractive as drug targets against these pathogens.

## Author Contributions

RE, VS, and E-MF developed the ideas for the manuscript, and wrote and read the manuscript.

## Conflict of Interest Statement

The authors declare that the research was conducted in the absence of any commercial or financial relationships that could be construed as a potential conflict of interest.
